# Abdominal obesity in COPD is associated with specific metabolic and functional phenotypes

**DOI:** 10.1186/s12986-022-00714-z

**Published:** 2022-12-01

**Authors:** Clayton L. Cruthirds, Nicolaas E. P. Deutz, Yani G. G. Mizubuti, Rajesh I. Harrykissoon, Anthony J. Zachria, Mariëlle P. K. J. Engelen

**Affiliations:** 1 Center for Translational Research in Aging and Longevity, Department of Kinesiology and Sport Management, Texas A&M University, 675 John Kimbrough Blvd, College Station, TX 77840 USA; 2 Pulmonary, Critical Care and Sleep Medicine, Scott and White Medical Center, College Station, TX USA

**Keywords:** Abdominal obesity, COPD, Branched-chain amino acids, Metabolism

## Abstract

**Background:**

Abdominal obesity (AO) is linked to reduced health status and mortality. While it is known that AO is prevalent in chronic obstructive pulmonary disease (AO-COPD), the specific metabolic and functional consequences associated with AO-COPD remain understudied.

**Methods:**

We studied 199 older adults with COPD and 168 control subjects with and without AO and assessed visceral adipose tissue (VAT) by dual-energy X-ray absorptiometry. VAT > 70th percentile of the control group qualified a subject as AO in a sex specific manner. We measured plasma concentrations and whole body production (WBP) rates of multiple amino acids to assess the metabolic profile. We assessed medical history, body composition by Dual-Energy X-ray Absorptiometry, muscle strength, and cognitive function. We performed statistics by analysis of covariance (p) and FDR (q) for multiple comparisons.

**Results:**

AO-COPD subjects had 27% more VAT (q < 0.01) than AO-Control subjects despite correction for BMI. Branched-chain amino acid concentrations and WBP rates were generally elevated in AO-COPD but whole body clearance rate was only elevated in COPD. Metabolic syndrome comorbidities (*p* < 0.01) and systemic inflammation (*P* < 0.05) were most prevalent in the AO-COPD group. Muscle strength was reduced in COPD subjects (*p* < 0.001), but partially preserved when combined with AO. Cognitive dysfunction and mood disturbances were present in COPD subjects (*p* < 0.001) with worst performers in AO-COPD (q < 0.05).

**Conclusion:**

The presence of AO is associated with specific metabolic and functional phenotypes in COPD.

*Clinical trial registry* Trial registration ClinicalTrials.gov. In the present paper, we report an analysis of the baseline measurements of COPD subjects and healthy controls from the study numbers: NCT01787682, NCT01787682, NCT02157844, NCT02082418, NCT02065141, NCT02770092, NCT02908425, NCT03159390, NCT02780219, NCT03327181, NCT03796455, NCT04928872, NCT04461236, NCT01173354, NCT01154400.

**Supplementary Information:**

The online version contains supplementary material available at 10.1186/s12986-022-00714-z.

## Background

Abdominal obesity (AO) is observed in 75% of patients with Chronic Obstructive Pulmonary Disease (COPD) [1] and associated with comorbidities such as hypertension, dyslipidemia, and diabetes [2]. AO is a significant contributor to the enhanced systemic inflammatory state and increased mortality in COPD subjects [1, 3]. The prevalence of AO is higher in COPD patients in comparison to non-COPD controls with similar BMI values [3].

The effects of AO on metabolism in COPD are underexplored. In conditions characterized by excess fat mass such as obesity and diabetes, the plasma concentrations of the branched-chain amino acids leucine, valine, and isoleucine (BCAA) are increased [4]. We and others previously observed higher plasma BCAA concentrations in COPD patients with preserved or elevated body weight [5, 6]. It has been suggested that elevated plasma BCAA concentrations in obesity are reflective of a dysregulation in BCAA and related glutamate metabolism [7], as plasma glutamate concentration was strongly correlated with visceral adipocyte diameter and an independent predictor of visceral adipose tissue (VAT) area [2]. However, plasma concentrations do not always reflect very well dysregulation of metabolism [8]. To gain more insight into the mechanisms underlying the elevated BCAA plasma concentrations in COPD, we need to measure whole-body production and clearance rates of leucine, valine, isoleucine, and glutamate using stable isotope tracer methodologies.

When COPD patients have a preserved muscle mass [9, 10], the presence of AO is associated with better muscle function (e.g., leg muscle strength and cycling performance). Only with low muscle mass (i.e., sarcopenia) is 6-min walk test performance worse in AO-COPD [10, 11]. In addition, AO in older adults is associated with cognitive impairment [12], but the relationship between cognitive decline and AO in COPD remains unclear [13]. However, abdominal obesity in COPD is often associated with the presence of metabolic syndrome which has implications on functional and clinical outcomes [14].

We, therefore, studied whether AO in COPD is associated with specific metabolic and functional phenotypes measured in a large group of freely-living, COPD patients and control subjects. Assessments included plasma concentrations and kinetics of amino acids with a special focus on the BCAA and glutamate, and their interactions, body composition, disease characteristics (e.g., lung function, comorbidities, systemic inflammation, glucose), muscle and cognitive function, and lifestyle factors (e.g., physical activity and dietary intake). VAT was assessed by dual-energy X-ray absorptiometry as it is the most direct assessment of abdominal fat mass compared to other available techniques (e.g., waist circumference, android/gynoid ratio). We hypothesize that AO in COPD is associated with preserved muscle mass but impaired muscle and cognitive function due to the enhanced systemic inflammatory response and specific disturbances in BCAA metabolism related to a high prevalence of metabolic syndrome in this subgroup of COPD patients.

## Methods

### Subjects

**Fig. 1:**
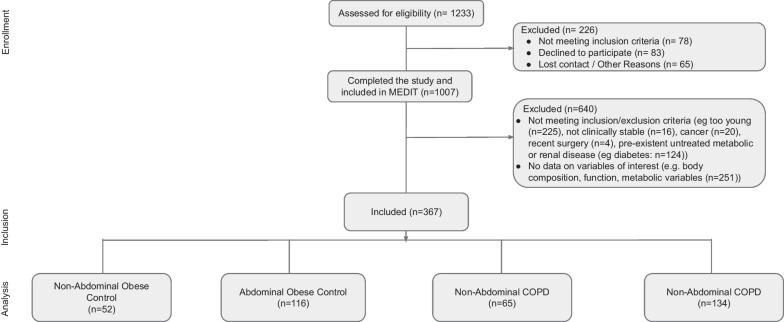
Flow chart of the subjects enrolled and studied

We studied participants who reported to the lab as part of the MEDIT (MEtabolism of Disease with Isotope Tracers) trial for all study outcomes (Fig. 1). MEDIT is a large controlled trial between 2013 and 2020 in healthy and diseased subjects who were well characterized by their skeletal muscle health (strength and mass), body composition, and comprehensive metabolic characterization by the combined pulse of stable tracers of multiple amino acids. Recruitment took place through pulmonologist referral and via advertisements in the local community. The MEDIT trial combines the baseline measurement of prospective trials. In this study, we used baseline values of COPD subjects and healthy controls of several registered trials (NCT01787682, NCT01787682, NCT02157844, NCT02082418, NCT02065141, NCT02770092, NCT02908425, NCT03159390, NCT02780219, NCT03327181, NCT03796455, NCT04928872, NCT04461236, NCT01173354, NCT01154400) using a "subject type" as a primary selector and data availability on metabolic data as a secondary selector. The "subject type" referred to the studied population in a given clinical trial and was coded based on extensive screening, including physical examination and medical history interview. We selected subjects who were ≥ 55 years old, ability to walk and stand up independently, and availability of DEXA data (for AO quantification). Diagnosis of COPD was based on an FEV1/FVC < 70% [15] or confirmation from referring physician/medical history. All selected COPD patients were clinically stable and did not suffer from an exacerbation or any infection of the respiratory tract ≤ 4 weeks before the study. Medical history and medication use were assessed as part of the screening process to assess comorbidities. Exclusion criteria for COPD and control subjects were pre-existent untreated metabolic or renal disease (including diabetes mellitus requiring daily insulin administration), malignancy, recent surgery, and use of systemic corticosteroids (< 4 weeks before the study day). We studied 199 older adults with COPD (GOLD 1–4) [15] and 168 control subjects (Table 1). Written informed consent was obtained from all subjects, and the study was approved by the Institutional Review Board of Texas A&M University.

**Table 1:** Subject characteristics, pulmonary function, and body composition

	Non-AO control (n = 52)	AO-control (n = 116)	Non-AO COPD (n = 65)	AO-COPD (n = 134)	ANCOVA *p*-values
Age (years)	67.3^a^ [67.0, 67.6]	69.1^b^ [68.7, 69.5]	68.3^c^ [67.9, 68.7]	68.6^c^ [68.2, 69.0]	C: 0.9095 **AO: 0.0007**
Male %	28/54%	46/40%	31/48%	63/47%	
*Pulmonary function and COPD related measures*					
FEV_1_ (% of predicted)	103.0^a^ [100.9, 105.1]	94.0^b^ [91.7, 96.2]	34.7^c^ [33.7, 35.6]	42.9^d^ [42.2, 43.5]	**C: 0.0001** AO: 0.1087 C*AO: 0.0015
FVC (% of predicted)	96.2^a^ [95, 97.3]	86.2^b^ [85.3, 87]	52.7^c^ [51.9, 53.5]	57.3^d^ [56.9, 57.7]	**C: 0.0001** AO: 0.1664 C*AO: 0.0002
FEV_1_/FVC (ratio)	79.7^a^ [78.9, 80.5]	84.0^b^ [82.7, 85.3]	50.0^c^ [49.2, 50.8]	55.9^d^ [55.3, 56.6]	**C: < 0.0001** AO: 0.5189
Oxygen saturation (%)	97.5^a^ [97.4, 97.7]	97.4^b^ [97.3, 97.5]	95.4^c^ [95.3, 95.5]	95.2^d^ [95.1, 95.3]	**C: < 0.0001** AO: 0.4186
mMRC dyspnea scale	N/A	N/A	2.35^a^ [2.07, 2.64]	2.32^a^ [2.03, 2.44]	–
Gold stage (%; 1/2/3/4)	N/A	N/A	2/31/35/32	8/29/43/20	–
Oxygen users (% yes)	N/A	N/A	48%^a^	48%^a^	–
Smoking status (non-smoker/former smoker/current smoker) (%)	96/0/4%	72/3/25%	8/29/63%	17/15/68%	–
Smoking history (pack years)	6.4^a^ [4.8, 8.0]	6.6^a^ [5.6, 7.6]	41.5^b^ [39.6, 43.4]	44.0^c^ [43.1, 44.8]	**C: < 0.0001** AO: 0.6105
No. of exacerbations in past year	N/A	N/A	0.68^a^ [0.66, 0.7]	0.66^b^ [0.65, 0.67]	–
No. of hospitalizations in last year for exacerbation	N/A	N/A	0.25^a^ [0.23, 0.26]	0.30^b^ [0.29, 0.31]	–
Short-acting bronchodilator (% yes)	N/A	N/A	61%^a^	55%^a^	–
Long-acting bronchodilator (% yes)	N/A	N/A	43%^a^	60%^a^	–
Inhaled corticosteroids (% yes)	N/A	N/A	59%^a^	50%^a^	–
Oral corticosteroids (% yes)	N/A	N/A	14%^a^	7%^a^	–
PASE (score)	161.1^a^ [156.3, 165.8]	169.4^b^ [164.4, 174.4]	106.7^c^ [102.7, 110.8]	118.1^d^ [115.6, 120.7]	**C: < 0.0001** AO: 0.8935
Total caloric intake (kcal/day)	1848^a^ [1776, 1920]	1747^b^ [1698, 1796]	1859^a^ [1800, 1917]	1765^b^ [1738, 1792]	C: 0.9203 AO: 0.8222
Daily carbohydrate intake (g/day)	204.5^a^ [197.5, 211.5]	184.6^b^ [179, 190.3]	210.8^c^ [203.1, 218.6]	173.0^d^ [170.0, 175.9]	C: 0.6235 AO: 0.8575
Daily fat intake (g/day)	67.6^a^ [64, 71.2]	64.3^a^ [61.8, 66.7]	74.6^b^ [71.3, 77.9]	70.5^c^ [69, 71.9]	C: 0.3157 AO: 0.9933
Daily protein intake (g/day)	68.2 [64.3, 72]	73.9 [70.7, 77.2]	65.5 [62.7, 68.3]	70.1 [68.3, 71.9]	C: 0.3868 **AO: 0.0460**
*Body composition and metabolic syndrome characteristics*					
Height (m)	1.68 [1.66, 1.70]	1.66 [1.65, 1.67]	1.67 [1.65, 1.68]	1.66 [1.65, 1.67]	C: 0.2382 AO: 0.3370
Body weight (kg)	71.2^a^ [68.4, 74.1]	83.3^b^ [80.6, 86.1]	61.7^c^ [58.4, 65.0]	88.5^d^ [85.5, 91.4]	**C: 0.0001** **AO: 0.0005** C*AO: 0.0078
Body Mass Index (kg/m^2^)	24.8^a^ [24.4, 25.2]	29.9^b^ [29.6, 30.2]	22.0^c^ [21.5, 22.4]	31.7^d^ [31.4, 32]	C: 0.0736 **AO: < 0.0001** C*AO: < 0.0001
Total lean mass (kg)	47.7^a^ [45.3, 50.2]	48.7^a^ [47.1, 50.4]	41.6^b^ [39.2, 44]	50.8^c^ [49.1, 52.5]	**C: < 0.0001** AO: 0.7139 C*AO: 0.0217
Total fat mass (kg)	18.4^a^ [17.8, 19.1]	30.5^b^ [28.8, 32.2]	16.3^c^ [15.4, 17.2]	33.0^d^ [31.3, 34.7]	C: 0.7180 **AO: < 0.0001**
Android fat (%)	27.5^a^ [26.9, 28]	40.4^b^ [39.4, 41.4]	22.4^c^ [21.7, 23.2]	42.6^d^ [41.4, 43.7]	**C: 0.0077** **AO: < 0.0001** C*AO: 0.0041
Gynoid fat (%)	29.9^a^ [28.6, 31.2]	37.4^b^ [36.2, 38.6]	29.5^a^ [28.6, 30.4]	39.1^c^ [37.8, 40.3]	C: 0.1945 **AO: 0.0004**
Android/Gynoid % fat mass ratio	0.94^a^ [0.90, 0.97]	1.10^b^ [1.07, 1.12]	0.77^c^ [0.74, 0.81]	1.11^b^ [1.08, 1.14]	**C: < 0.0001** **AO: < 0.0001** C*AO: 0.0003
Heart rate (BPM)	64.0^a^ [63.3, 64.7]	67.4^b^ [66.9, 67.9]	74.0^c^ [73.4, 74.6]	72.0^d^ [71.5, 72.5]	**C: < 0.0001** AO: 0.6142 C*AO: 0.0420
Systolic blood pressure (mmHg)	125.4^a^ [124.4, 126.5]	137.3^b^ [136.3, 138.2]	131.1^c^ [129.5, 132.7]	134.2^d^ [133.4, 134.9]	C: 0.4272 AO: 0.8559 C*AO: 0.0076
Diastolic blood pressure (mmHg)	74.8^a^ [74.6, 75.0]	79^b^ [78.6, 79.3]	77.1^c^ [76.7, 77.4]	76.2^d^ [75.9, 76.5]	C: 0.9796 AO: 0.5630 C*AO: 0.0060
Charlson comorbidity index (score)	0.28^a^ [0.22, 0.33]	0.38^b^ [0.35, 0.42]	1.63^c^ [1.55, 1.71]	1.88^d^ [1.84, 1.92]	**C: < 0.0001** AO: 0.8473
Hypertension (% yes)	33%^a^	43%^a^	35%^a^	66%^b^	–
Dyslipidemia (% yes)	36%^a^	31%^a^	31%^a^	58%^b^	–
Obstructive sleep apnea (% yes)	5%^a^	29%^b^	14%^a^	41%^c^	–
Diabetes (% yes)	3%^a^	7%^a^	8%^a^	19%^b^	–

Data are presented as estimated mean [95% CI] unless otherwise stated. Statistics are by Fisher’s exact test or ANCOVA with multiple comparisons. ANCOVA was data as the dependent variable with as dummy variables COPD, AO, COPD*AO interaction, and confounders gender, age, and BMI. ANCOVA *p*-values are effects of COPD, AO, or COPD*AO from the model. Interaction effects were tested for all variables with only significant effects being included; bold is *p* < 0.05. Multiple comparisons are listed as superscript letters, same letters meaning no difference; q < 0.05. Oxygen and medication use and prevalence of comorbidities were tested via Fisher’s exact test

FEV_1_, Forced Expiratory Volume in one second. FVC, Forced Vital Capacity. mMRC, Modified Medical Research Council. PASE, physical activity questionnaire for the elderly

### Anthropometrics, body composition, and disease characteristics

We measured body weight and height by a digital beam scale and stadiometer, and regional values for fat mass and fat-free mass in the supine position by Dual-Energy X-ray Absorptiometry (DXA) (Hologic QDR 4500/ Version 12.7.3.1, Hologic, Bedford, USA). DXA was performed by the same researcher according to lab standard operating procedures to assure consistency.

Despite the fact that some studies have investigated the association between excess VAT and the association with cardio-metabolic risk, few have proposed VAT cutoff points using DXA [16]. We based the definition of AO on visceral adipose tissue (VAT) cutoffs obtained from the control group as reported from DXA. VAT 70th [17] or higher percentile of the control group qualified a subject as AO in a sex- specific manner (> 737 g M; > 485 g F). If a subject was below this cutoff they were considered non-abdominally obese (non-AO). We studied 65 non-AO and 134 AO-COPD patients, and 52 non-AO and 116 AO-control subjects.

We used spirometry to measure Forced Vital Capacity (FVC) and Forced expiratory volume in 1 s (FEV_1_) and used the highest value from ≥ 3 technically acceptable maneuvers [18]. We used the modified medical research council dyspnea scale (mMRC) to assess the level of dyspnea, and Charlson Comorbidity Index [19] for assessment of concomitant comorbidities. Postabsorptive plasma was sampled in each subject for analysis of glucose and the inflammatory marker high-sensitivity C-reactive protein (hs-CRP) on a Cobas C111 Analyzer with standard kits (Roche Diagnostics, Mannheim, Germany).

### Isotope administration and calculations

We placed a peripheral line in a superficial dorsal vein of the hand or lower arm for a pulse infusion of a cocktail of stable amino acid tracers and subsequent blood sampling (Cambridge Isotope Laboratories, Woburn, USA) [20, 21]. We placed the hand and lower arm in a thermostatically controlled hot box (internal temperature: 60 °C), a technique to mimic direct arterial sampling [22]. After a blood sample was collected to measure baseline enrichment, the bolus tracer infusion was given containing a cocktail of 17 different amino acid isotopes including those related to BCAA, protein, and miscellaneous metabolic pathways [20, 21]. We sampled arterialized-venous blood at multiple time points until two hours after pulse administration.

We put arterialized-venous blood in Li-heparinized (Becton Dickinson Vacutainer system, Franklin Lakes, USA), immediately on ice to minimize enzymatic reactions, and centrifuged to obtain plasma. We aliquoted part of the plasma into tubes with trichloroacetic acid for deproteinization purposes and immediately frozen and stored them at − 80 °C until further analysis. We measured tracer enrichments [tracer: tracee ratio (TTR)] and plasma amino acid concentrations batch-wise by Liquid Chromatography tandem Mass Spectrometry (LC–MS/MS) [20] and calculated whole body production (WBP) rates of amino acids [20].

### Muscle and cognitive function and questionnaires

A single leg muscle strength test was completed at 60°/s with the right limb, using an isokinetic dynamometer (Isokinetic International, Chattanooga, USA) [23]. Briefly, after a warm-up (10 low-effort repetitions), peak leg torque and force were assessed by 5 maximal extension-flexion cycles, each cycle followed by 10 s of rest. We assessed maximal expiratory pressure (MEP) and inspiratory pressure (MIP) as measures of respiratory muscle strength by determining the maximal value of at least 3 reliable attempts using a hand-held mouth pressure device (Micro Respiratory Pressure Meter (RPM)) with at least 1 min of rest between each attempt [24].

We used the Trail Making Test (TMT) to assess visual-motor tracking skills and psychomotor speed [25]. In brief, the subjects connect consecutive numbers randomly arranged on a page (TMT part A) or consecutive numbers and letters in alternating order (TMT part B). We used the Stroop Color-Word Test (SCWT) to measure executive functioning and cognitive flexibility as response inhibition for colored printed words across three parts [23] and completion times (s) were recorded for each part.

We assessed mood status (depression and anxiety) by the “Hospital Anxiety and Depression Scale” (HADS) [26] and habitual dietary intake using 24-h dietary recall while daily physical activity level was measured by the “Physical Activity Scale for the Elderly” questionnaire (PASE).

### Statistical analysis

Raw data was checked for normality by the likelihood ratio of sampling from a Gaussian or a lognormal distribution. If data failed the normality test, we log-transformed and performed statistics on those data. We calculated group effects of continuous variables with analysis of covariance (ANCOVA). As dummy variables, we used the presence of COPD and AO with confounders’ sex, age, and BMI. COPD*AO interaction was tested for all comparisons and only included if significant. Estimated values from the ANCOVA model were used for all tables and graphs. We tested multiple comparisons on estimated values by controlling the false discovery rate [27] using the two-stage step-up method of Benjamini, Kreiger, and Yekutieli [28], providing the false discovery rate-adjusted *p*-value as q-value. Discrete variables are presented as percentages and compared using Fisher's exact test. We have set the level of significance at p or q < 0.05 and used the statistical package Graphpad Prism (Version 9.0.0, GraphPad Software Inc, San Diego, USA) for data analysis.

## Results

### General and disease characteristics and body composition

We studied 199 older adults with a clinical diagnosis of COPD (65 non-AO, 134 AO) and 168 control subjects (52 non-AO, 116 AO). AO subjects were older than the non-AO (*p* = 0.001), independent of the presence of COPD (Table 1). Lung function was reduced in COPD subjects (*p*<0.001) but more preserved in those with AO. Physical activity level was reduced in COPD subjects (*p* < 0.001) but independent of the presence of AO, and higher daily protein intake in AO subjects (*p* = 0.046) was the only difference in dietary intake across all groups (Table 1).

**Fig. 2:**
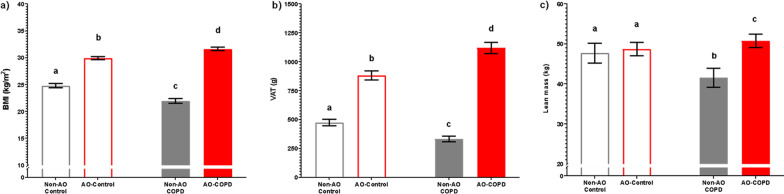
Values are estimated mean [95% CI]. *P*-values are effects of COPD, AO, or COPD*AO from the ANCOVA model. Interaction effects were tested for all variables with only significant effects being included, *P* < 0.05. Multiple comparisons are displayed with letters, same letters meaning no difference, q < 0.05. **a** Body Mass Index (BMI); C: *P* = 0.0736, AO: *P* < 0.001, C*AO: *P* < 0.001. **b** Visceral Adipose Tissue (VAT); C: *P* = 0.1299, AO: *P* < 0.001, C*AO: *P* < 0.001. **c** Total lean mass; C: *P* < 0.001, AO: *P* = 0.7139, C*AO: *P* = 0.0217

**Fig. 3:**
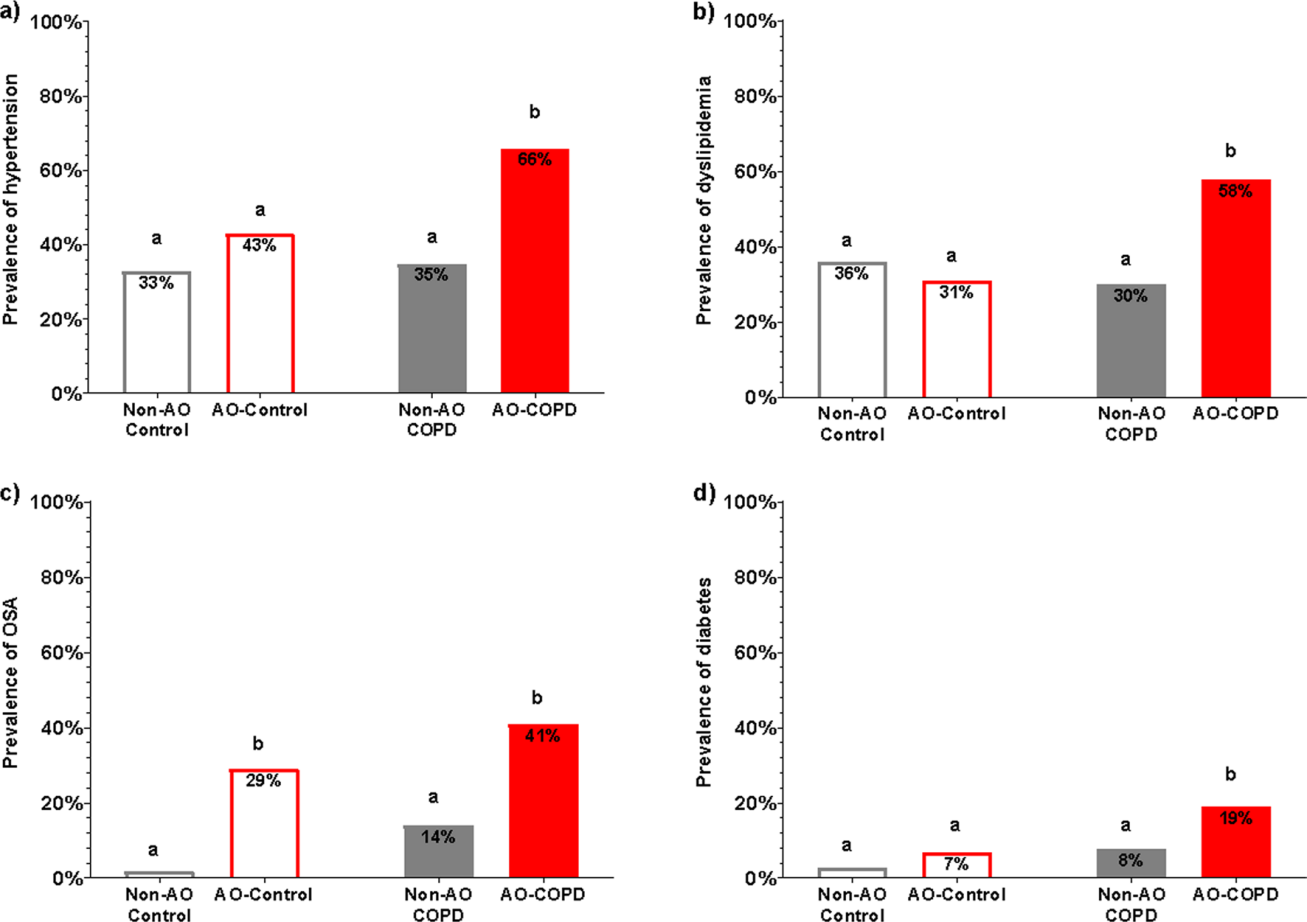
Values are percent prevalence of comorbidity. Differences were tested using Fisher's exact test and are displayed with letters, same letters meaning no difference, *p* < 0.05. **a** Prevalence of hypertension. **b** Prevalence of dyslipidemia. **c** Prevalence of Obstructive Sleep Apnea. **d** Prevalence of Diabetes

**Fig. 4:**
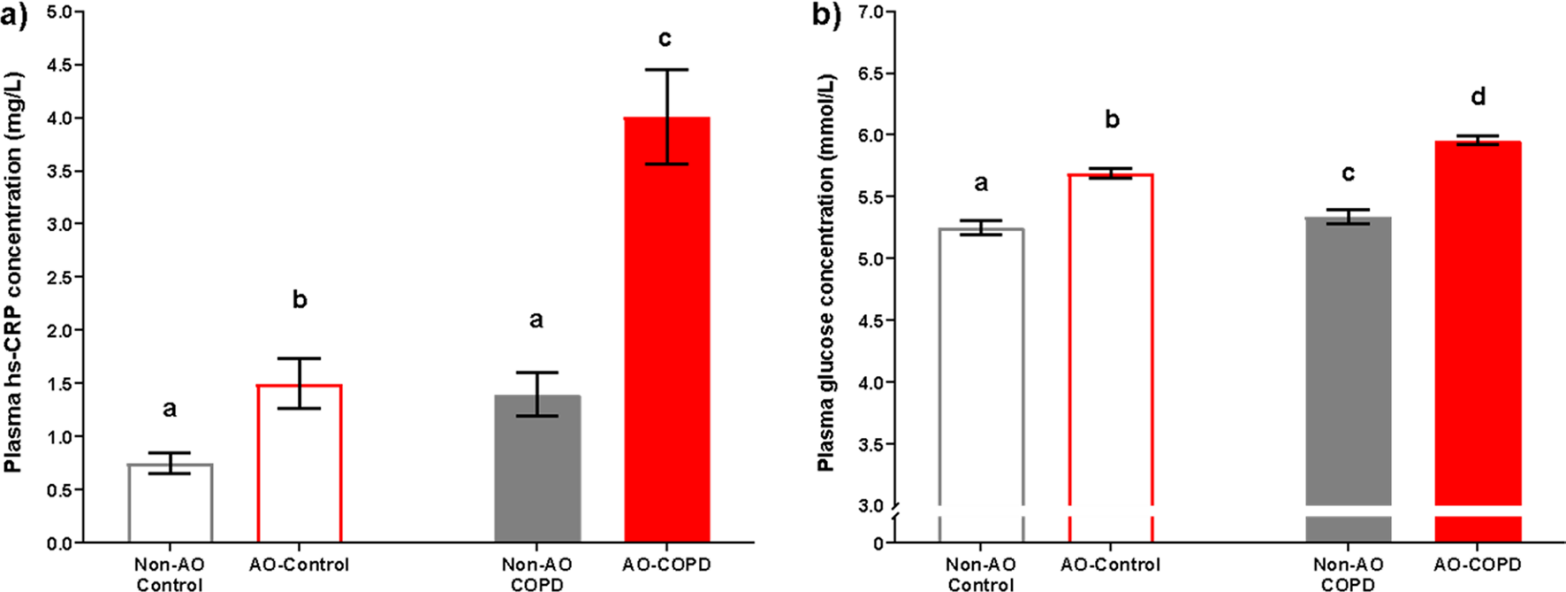
Values are estimated mean [95% CI]. *P*-values are effects of COPD, AO, or COPD*AO from the ANCOVA model. Interaction effects were tested for all variables with only significant effects being included, *P* < 0.05. Multiple comparisons are displayed with letters, same letters meaning no difference, q < 0.05. **a** Plasma hs-CRP concentration; C: *P* < 0.001, AO: *P* = 0.3342. **b** Plasma glucose concentration; C: *P* = 0.1121, AO: *P* = 0.0500

BMI and VAT were elevated in AO subjects (*p* < 0.001) with COPD*AO interactions (*p* < 0.001, *p* < 0.001) (Fig. 2a, b, Table 1). BMI and VAT were highest in the AO-COPD group compared to all other groups (q < 0.05). The AO-COPD group had 27% more VAT than AO-Control despite a comparable A/G ratio (Table 1). Lean mass was lower in COPD subjects (*p* < 0.001) with a COPD*AO interaction (*p* = 0.022) such that it was specifically preserved in AO-COPD (q < 0.05) but reduced in the non-AO COPD (Fig. 2c, Table 1). The composite Charlson Comorbidity Index score was elevated in COPD subjects (*p* < 0.001), with the highest scores in the AO-COPD group (q < 0.05) (Table 1). Metabolic syndrome-related comorbidities such as hypertension, dyslipidemia, diabetes, and obstructive sleep apnea (OSA) were most prevalent in the AO-COPD group (*p* < 0.01) (Fig. 3a–d, Table 1). Plasma hs-CRP concentrations were elevated in COPD subjects (*p* < 0.001) with the highest values in the AO-COPD group (q < 0.05) (Fig. 4a). Plasma glucose concentrations tended to be elevated in AO subjects (*p* = 0.050) (Fig. 4b).

### Metabolic characteristics

**Fig. 5:**
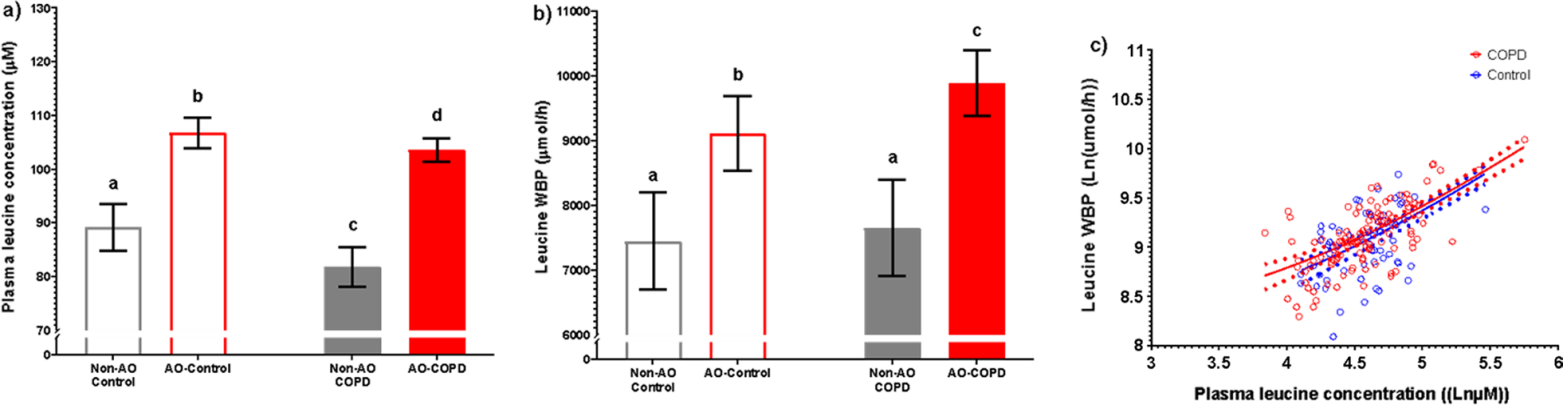
Values are estimated mean [95% CI] (**a**, **b**) or Ln(value) with regression line [95% CI] (**c**). *P*-values are effects of COPD, AO, or COPD*AO from the ANCOVA model. The model for panel (**c**) additionally included plasma leucine concentration. Difference in the slopes were tested, if no difference a shared slope was applied. Interaction effects were tested for all variables with only significant effects being included, *P* < 0.05. Multiple comparisons are displayed with letters, same letters meaning no difference, q < 0.05. **a** Plasma leucine concentration; C: *P* = 0.2029, AO: *P* < 0.001. **b** Leucine whole body production; C: *P* = 0.0616, AO: *P* = 0.2765. **c** Leucine clearance; C: *P* = 0.0328, AO *P* = 0.4927

Plasma concentrations of leucine, valine, and isoleucine were elevated in AO subjects (*p* < 0.001) (Fig. 5a, Additional file 1: Table S1). These elevations were particularly present in the AO-COPD group (q < 0.05), whereas the plasma concentrations for leucine and valine in the non-AO COPD were reduced. Plasma glutamate (*p* = 0.001) and alanine (*p* = 0.047) concentrations were also elevated in AO subjects and highest in the AO-COPD group (q < 0.05) (Additional file 1: Table S1). Additionally, sum of the essential amino acids (*p* = 0.001: EAA) was elevated in AO subjects with a COPD*AO interaction (*p* = 0.029), with the highest values in the AO-COPD group and the lowest in the non-AO COPD group (Additional file 1: Table S1). Comparisons of remaining plasma amino acid concentrations are described in Additional file 1: Table S1.

Of the three BCAA, WBP of valine was generally elevated in AO subjects (*p* = 0.017) (Additional file 1: Table S3) with WBP of leucine and isoleucine being highest in AO-COPD (q < 0.05) (Fig. 5b, Additional file 1: Table S3). Multiple comparisons revealed WBP of glutamate was highest in AO-Control subjects specifically (q < 0.05) (Additional file 1: Table S3). WBP of phenylalanine, representative of protein breakdown, was elevated in AO subjects (*p* = 0.026) (Additional file 1: Table S3). WBP rates of the amino acids hydroxyproline (marker of collagen breakdown) and taurine were elevated in COPD (*p* = 0.032, *p* < 0.001, resp.) and highest in the AO-COPD group (q < 0.05) (Additional file 1: Table S3). Group comparisons of remaining WBP rates are described in Additional file 1: Table S3.

In order to assess whole-body disposal rates of the amino acids, we examined the association between WBP rate and plasma concentration, representative of clearance rate. The clearance rate of leucine was elevated in COPD subjects (*p* = 0.033) with no effect of AO or interactions (Fig. 5c, Additional file 1: Table S3). The clearance rate of phenylalanine was reduced (*p* = 0.033) while taurine was elevated (*p* < 0.001) in COPD (Additional file 1: Table S3). The remaining amino acid clearance rates are described in Additional file 1: Table S3.

### Muscle and cognitive function and questionnaires

**Fig. 6:**
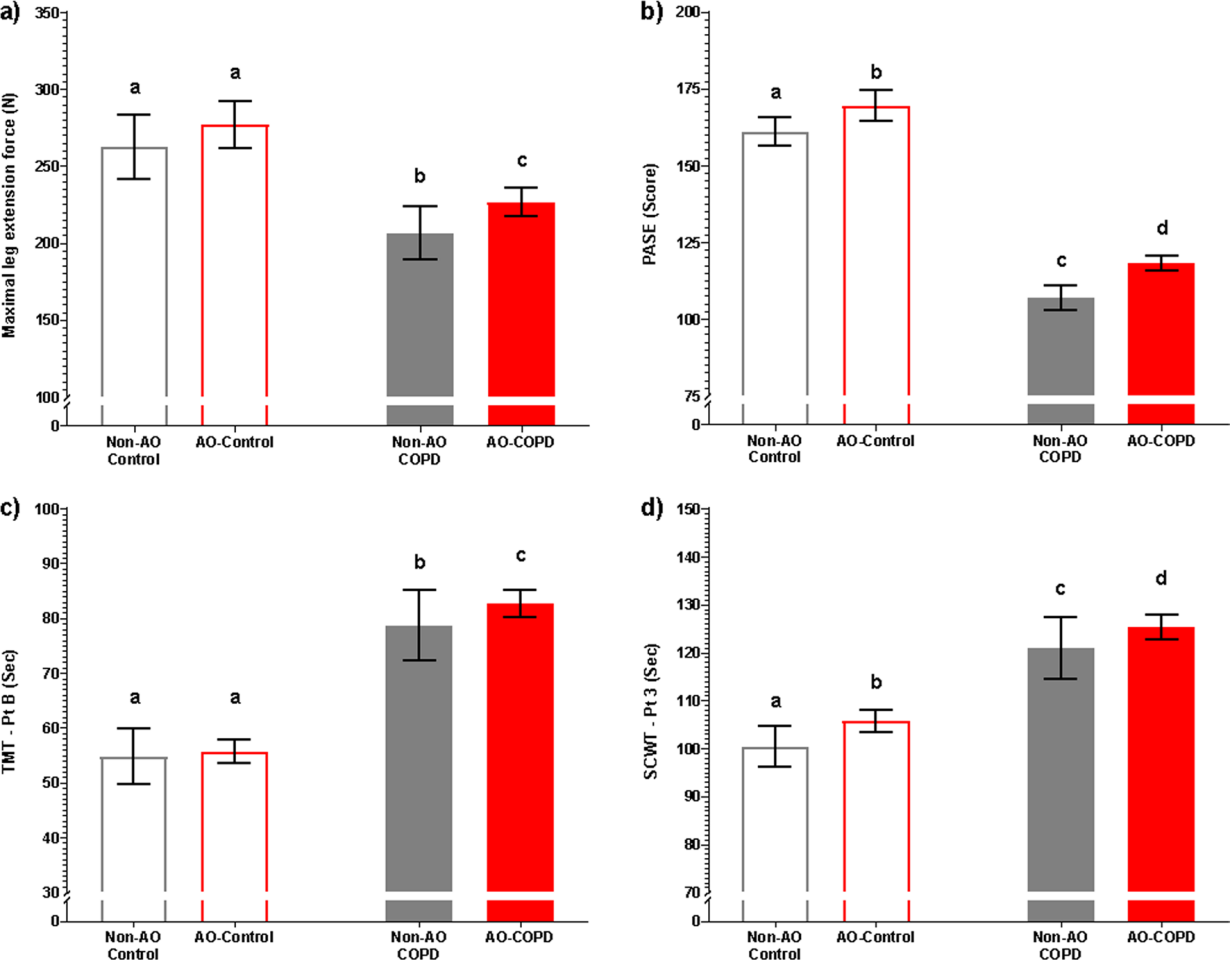
Values are estimated mean [95% CI]. *P*-values are effects of COPD, AO, or COPD*AO from the ANCOVA model. Interaction effects were tested for all variables with only significant effects being included, *P* < 0.05. Multiple comparisons are displayed with letters, same letters meaning no difference, q < 0.05. **a** Maximal leg extension force; C: *P* < 0.001, AO: *P* = 0.0625. **b** PASE score; C: *P* < 0.001, AO: *P* = 0.8935. **c** TMT-Pt B; C: *P* < 0.001, AO: *P* = 0.7867. **d** SCWT-Pt 3; C: *P* < 0.001, AO: *P* = 0.2190

Leg muscle strength and physical activity level were reduced in the COPD group (*p* < 0.001, *p* < 0.001, resp.) but more preserved in those with AO (Fig. 6a, b, Additional file 1: Tables S1–S4). Similarly, inspiratory (*p* < 0.001) and expiratory muscle strength (*p* < 0.001) were lower in COPD subjects with COPD*AO interactions such that strength was more preserved in AO-COPD subjects (*p* = 0.004, *p* = 0.005, resp.) (Additional file 1: Table S4). Cognitive function was impaired across all parts of the TMT and SCWT in COPD subjects (*p* < 0.001) (Fig. 6c, d, Additional file 1: Table S4). Multiple comparisons revealed AO-COPD subjects generally had the worst performance compared to all other groups (q < 0.05). Additionally, anxiety and depression were elevated in COPD subjects (*p* < 0.001, *p* < 0.001, resp.) with the highest values in those with AO (Additional file 1: Table S4).

## Discussion

We tested whether COPD patients with abdominal obesity have a specific metabolic and/or functional phenotype by studying a large group of free-living, older adults. The COPD patients with AO had a higher VAT than control subjects despite correction for differences in age, sex, and BMI. Plasma BCAA concentrations and whole body production rates, but not clearance, were increased in AO subjects. Although lean mass, muscle function, and physical activity level were lower in COPD patients, these were relatively preserved in those with AO. However, AO-COPD was also characterized by elevations in systemic inflammation, metabolic syndrome-related comorbidities, cognitive dysfunction, and mood disturbances.

Classically COPD patients were divided into two phenotypes, the “pink puffer” and “blue bloater” [29], the former being characterized by emphysema and wasting of both muscle and fat tissue [6, 30]. The studied non-AO COPD phenotype, present in 32% of our COPD group, showed comparable characteristics as reflected by lower values for BMI, lean mass, muscle strength, concentrations of BCAA, and total essential amino acids as compared to non-AO controls and AO-COPD and more impaired lung function. Cognition and wellbeing were reduced in non-AO COPD as compared to non-AO Controls. In line, we have previously shown that COPD patients with low lean mass have muscle dysfunction [21, 31] and are characterized by reduced BCAA concentrations [6]. High atherosclerotic cardiovascular disease risk has been found to be significantly associated with sarcopenia in men with COPD, particularly in those with moderate to very severe airflow limitation, independent of central obesity and fat mass [32]. No increased prevalence of hypertension or dyslipidemia was observed in the non-AO group, although this group had higher prevalence of OSA and diabetes as compared to the non-AO controls. Despite the lower prevalence of this phenotype in our outpatient COPD population, the sarcopenic phenotype is still apparent and should be targeted with specific interventions. Previous studies showed that COPD patients respond anabolic to dietary essential amino acids [33]. Our data suggest that these patients would most benefit from resistance type exercise training combined with essential amino acid enriched oral nutritional supplementation to increase their muscle mass and function.

Sarcopenic obesity is a clinical and functional condition characterized by the coexistence of excess fat mass and sarcopenia [34]. Currently, different definitions of sarcopenic obesity exist and its diagnostic criteria and cut-offs are not universally established. Therefore, the prevalence and sensitivity of this condition for any disease risk prediction is affected significantly.

In the present study, we chose VAT as it is the most direct assessment of abdominal fat mass compared to other available techniques (e.g., waist circumference, android/gynoid ratio). AO-COPD had 27% more VAT compared to AO-Control, despite comparable values for android/gynoid ratio. This difference remained after correcting for the difference in BMI. This supports the use of VAT as a valuable assessment of AO in older subjects with and without chronic disease. Interestingly this VAT elevation in AO-COPD occurred despite lower self-reported carbohydrate and fat intake compared to the AO-Control subjects. The reason for this discrepancy is unknown. A second cause of the elevated VAT specifically in AO-COPD could be due to blunted IL-6 signaling caused by chronic activation of the inflammatory pathway, which has been shown to negate the reduction in VAT from exercise training [35]. Whether inflammation or increased VAT comes first in this cycle deserves further study. Besides CRP, also other signaling factors such as adipokines and leptin need to be further explored in these patients due to their roles as biomarkers in COPD [36]. Besides its role in body weight control, leptin may also act as a vasoactive hormone. Via changing the vascular responses of angiotensin II, leptin is involved in blood pressure regulation [37].

In general, plasma concentrations do not represent production or disposal of substrates [8]. For instance, we previously reported unchanged plasma BCAA concentrations despite elevated BCAA turnover in weight stable COPD patients [38]. Therefore we also measured turnover of the individual BCAA in the present study to obtain a more detailed insight in their metabolism. We now observed that elevations in plasma concentrations of BCAA in AO are met with an equal increase in turnover of BCAA, but that therefore their clearance rate is unchanged in AO subjects. In line with the higher prevalence of metabolic syndrome comorbidities such as hypertension, diabetes, and dyslipidemia observed in our AO-COPD group, higher baseline plasma BCAA concentrations are associated with an increased risk of cardiovascular disease [39] and insulin resistance and diabetes [4]. Dietary BCAAs intake has been shown to be associated with increased odds of general obesity, in particular among men. However, no significant association has been observed between dietary BCAAs and abdominal obesity [40].

Further research is needed to study why BCAA turnover is increased in AO.

Elevated plasma glutamate concentrations were generally seen in all AO subjects but were highest in the AO-Control group therefore not coinciding with the group with the highest VAT amount. This is in contrast to previously reported data of plasma glutamate concentration being an independent predictor of VAT area [2]. This could be reflective of an interactive effect of AO and COPD as muscle glutamate levels are reduced in COPD patients [41].

Some other observations further support COPD and AO as potent effectors of whole-body metabolism. For instance, elevations in plasma concentrations and WBP of hydroxyproline in our COPD patients possibly reflect previous data in inflammatory lung disease and could be indicative of enhanced whole body collagen breakdown [42]. The elevated WBP of taurine seen in all COPD patients and highest values in AO-COPD further the link between taurine metabolism and inflammatory diseases [43].

COPD patients with AO had partially preserved lung function over their non-AO counterparts. The AO patients also had fewer exacerbations in the past year despite more pack-years of smoking history. We also saw an elevated lean mass and partial preservation of skeletal and respiratory muscle function, and physical activity level in the AO-COPD group. Thus the presence of AO appears to have some clinical benefit to COPD patients. This improvement in muscle function has been shown previously [9, 10], with the largest strength improvement relative to sarcopenic COPD subjects [10]. Muscle quality (function relative to mass) was still impaired in AO-COPD. Future studies should focus on interventions specifically targeting further improvement in muscle function in AO-COPD patients. While various definitions of sarcopenia are used in the literature, a recent analysis has validated an age-sex-BMI specific cutoff in COPD patients [44]. With studies often focusing on the combination of sarcopenia and obesity [1, 10, 11], it would be of interest to examine this combined effect in our free-living COPD outpatients.

Despite these limited benefits of AO in COPD, there were clear detriments highlighted by cognitive dysfunction, anxiety, depression, and metabolic syndrome-related comorbidities. Cognitive dysfunction is common in the general COPD population [13] and older adults with AO [12]. However, the combined effects of AO and COPD have not been studied. Cognitive dysfunction in older adults was specifically linked to AO over simply being overweight [12]. Both these and our findings in AO remained after correction for BMI. Mood disturbances are a common comorbidity in COPD affecting up to 70% of COPD patients [45]. Conflicting evidence exists whether changes in body composition may influence depression as both an increase [46] and decrease in BMI [47] have been found to be associated with depression. Our data show that depression and anxiety are elevated in COPD, especially in those with AO. This increase remains after correcting for BMI thus supporting regional body composition as an important factor in cognitive dysfunction and mood disturbances.

We and others [48] found that COPD patients have more comorbidities, specifically those related to metabolic syndrome and that these are a large contributor to the increased all-cause and cardiovascular disease related mortality [49]. We, therefore, suggest that specifically AO-COPD patients should receive a targeted intervention. For instance, 12 weeks of cycling training can increase cardiovascular fitness and reduced VAT and cholesterol in healthy older adults [35] or daily supplementation with HMB, a metabolite of leucine, for four weeks can reduce VAT while maintaining muscle mass [50]. We hypothesize that aerobic exercise and targeted nutritional support will reduce VAT and subsequently reduce systemic inflammation and the burden of metabolic syndrome comorbidities.

Some limitations of the current study need to be considered. Because we conducted the study at our Clinical Research Unit, the studied COPD subjects were clinically stable sedentary outpatients with a high prevalence of overweight. Therefore, the current results cannot be generalized to the global COPD population including those experiencing weight loss, acute exacerbations, or participating in rehabilitation programs. Furthermore, 10% of the studied COPD subjects had evidence of congestive heart failure and were using beta blockers. It has previously been suggested that beta-blockers may have negative metabolic effects due to reduced glycemic control, masking of hypoglycemia, insulin resistance, and dyslipidemia [51]. Increased fat mass was observed in CHF using beta blockers [52], and long-term beta blockers may increase protein oxidation and therefore decrease the fat-free tissue [53]. Future studies are needed to unravel the metabolic and functional implications of use of beta-blockers in COPD. Self-reported 24-h dietary recall was used to assess habitual dietary intake which may have underreported the actual dietary intake of the subjects. The best approach for dietary assessment in older persons is dependent on the purpose of assessment and the abilities of the subjects [54], which need to be established in COPD.

## Conclusions

We elucidated the metabolic and functional phenotypes of COPD patients with and without AO in a group of free-living, older adults. The higher VAT in the AO-COPD group was associated with relatively preserved lean mass, muscle function, and physical activity level but also elevations in systemic inflammation, metabolic syndrome-related comorbidities, and cognitive dysfunction. However, alterations in BCAA metabolism caused in either AO or COPD alone could not fully explain these functional and clinical consequences in combined AO-COPD.

## Supplementary Information

**Additional file 1: Table S1**. Plasma amino acid concentrations. **Table S2**. Amino acid whole body production rates. **Table S3**. Whole body clearance estimates. **Table S4**. Muscle and cognitive function.

## Data Availability

The datasets used and/or analyzed during the current study are available from the corresponding author on reasonable request.

## Abbreviations

FEV_1_: Forced Expiratory Volume in one second
FVC: Forced Vital Capacity
mMRC: Modified Medical Research Council
PASE: Physical activity questionnaire for the elderly
BMI: Body Mass Index
DXA: Dual-energy X-ray absorptiometry
VAT: Visceral Adipose Tissue
AO: Abdominal Obesity
MIP: Maximal inspiratory pressure
MEP: Maximal expiratory pressure
TMT: Trail Making Test
SCWT: Stroop Color Word Test
HADS: Hospital Anxiety and Depression Scale
LC–MS/MS: Liquid Chromatography tandem Mass Spectrometry
BCAA: Branched-Chain Amino Acids
NEAA: Non-Essential Amino Acids
EAA: Essential Amino Acids
WBP: Whole Body Production
