# Supplementary material for: Abdominal obesity in COPD is associated with specific metabolic and functional phenotypes
Source: Nutr Metab (Lond). 2022 Dec 1;19:79. doi: 10.1186/s12986-022-00714-z (PMC9714145; doi:10.1186/s12986-022-00714-z)
Supplement: Supplementary file 1 — Additional file 1: Table S1. Plasma amino acid concentrations. Table S2. Amino acid whole body production rates. Table S3. Whole body clearance estimates. Table S4. Muscle and cognitive function. [file 12986_2022_714_MOESM1_ESM.docx]

| Additional file 1: Table S1. Plasma amino acid concentrations | | | | | |
| --- | --- | --- | --- | --- | --- |
|  | **Non-AO Control  (n=52)** | **AO-Control**  **(n=116)** | **Non-AO COPD (n=65)** | **AO-COPD (n=134)** | **ANCOVA**  **p-values** |
| **BCAA related amino acids** | | | | | |
| Leucine | 89.2^a^  [84.9, 93.5] | 106.8^b^  [103.9, 109.6] | 81.8^c^  [78.1, 85.5] | 103.6^d^  [101.4, 105.9] | C: 0.2029  **AO: <0.0001** |
| Valine | 149.2^a^  [143.5, 154.8] | 179.6^b^  [175.9, 183.3] | 136.7^c^  [131.8, 141.6] | 172.5^d^  [169.6, 175.4] | C: 0.0951  **AO: <0.0001** |
| Isoleucine | 47.9^a^  [45.4, 50.4] | 56.2^b^  [54.6, 57.8] | 47.3^a^  [45, 49.5] | 59^c^  [57.6, 60.3] | C: 0.2918  **AO: 0.0006** |
| Glutamate | 38.9^a^  [37, 40.9] | 53.0^b^  [51.5, 54.5] | 36.2^c^  [34.5, 38] | 50.9^d^  [49.8, 52.1] | C: 0.5247  **AO: 0.0003** |
| Alanine | 257.8^a^  [255.4, 260.1] | 301.2^b^  [297.9, 304.4] | 257.6^a^  [254.6, 260.7] | 313.6^c^  [310.8, 316.5] | C: 0.4061  **AO: 0.0472** |
| **Remaining amino acids** | | | | | |
| Glycine | 257.7^a^  [245.4, 269.9] | 213.2^b^  [207.6, 218.9] | 271.5^c^  [260.7, 282.3] | 203.6^d^  [199.2, 208] | C: 0.6218  **AO: 0.0080** |
| Phenylalanine | 43.4^a^  [42.5, 44.2] | 48.3^b^  [47.8, 48.8] | 41.8^c^  [41.2, 42.5] | 47.3^d^  [46.9, 47.7] | C: 0.2712  **AO: 0.0002** |
| Tau-methylhistidine | 4.1^a^  [3.9, 4.2] | 4.3^b^  [4.2, 4.4] | 4.4^b^  [4.2, 4.6] | 4.9^c^  [4.9, 5.0] | **C: 0.0438**  AO: 0.8491 |
| Histidine | 64.4  [64, 64.8] | 67.0  [66.6, 67.3] | 58.5  [58, 58.9] | 59.7  [59.5, 59.9] | **C: <0.0001**  AO: 0.0847 |
| Hydroxyproline | 10.1  [10.0, 10.2] | 11.3  [11.2, 11.3] | 12.0  [11.9, 12.1] | 13.5  [13.4, 13.6] | **C: 0.0142**  AO: 0.2230 |
| Taurine | 37.4^a^  [36.7, 38.2] | 39.5^b^  [39.1, 39.9] | 35.6^c^  [34.9, 36.3] | 36.7^d^  [36.4, 37.1] | **C: 0.0213**  AO: 0.2095 |
| Tryptophan | 33.7^a^  [33.0, 34.4] | 37.4^b^  [36.8, 38.0] | 32.2^c^  [31.4, 32.9] | 34.1^a^  [33.8, 34.5] | **C: 0.0093**  **AO: 0.0001** |
| Sum NEAA | 1559  [1552, 1565] | 1566  [1562, 1569] | 1553  [1545, 1560] | 1553  [1550, 1556] | C: 0.7967  AO: 0.7674 |
| Sum EAA | 734.8^a^  [716.7, 753.0] | 780.1^b^  [769.5, 790.7] | 637.7^c^  [621.8, 653.6] | 769.4^d^  [761.2, 777.5] | **C: 0.0052**  **AO: 0.0001**  **C*AO: 0.0288** |
| Data are presented as estimated mean [95% CI] in µM. Statistics are by ANCOVA with multiple comparisons. ANCOVA was data as the dependent variable with confounders COPD, AO, COPD*AO interaction, gender, age, and BMI. ANCOVA p-values are effects of COPD, AO, or COPD*AO from the model. Interaction effects were tested for all variables with only significant effects being included; bold is p<0.05. Multiple comparisons are listed as superscript letters, same letters meaning no difference; q<0.05. Sum NEAA = Sum of the non-essential amino acids aspartate, glutamate, asparagine, glutamine, serine, glycine, arginine, alanine, proline and tyrosine. Sum EAA = Sum of the essential amino acids threonine, valine, methionine, isoleucine, leucine, tryptophan, phenylalanine, histidine and lysine. | | | | | |

| Additional file 1: Table S2. Amino acid whole body production rates | | | | | |
| --- | --- | --- | --- | --- | --- |
|  | **Non-AO Control  (n=52)** | **AO-Control**  **(n=116)** | **Non-AO COPD (n=65)** | **AO-COPD (n=134)** | **ANCOVA**  **p-values** |
| **BCAA related amino acids** | | | | | |
| Leucine | 7452^a^  [6698, 8206] | 9117^b^  [8541, 9692] | 7662^a^  [6920, 8404] | 9897^c^  [9388, 10405] | C: 0.0616  AO: 0.2765 |
| Valine | 8118^a^  [7090, 9146] | 10958^b^  [10092, 11825] | 8395^a^  [7393, 9397] | 11038^c^  [10367, 11709] | C: 0.9980  **AO: 0.0165** |
| Isoleucine | 2422^a^  [2151, 2693] | 2885^b^  [2691, 3078] | 2795^b^  [2529, 3061] | 3464^c^  [3280, 3648] | C: 0.0506  AO: 0.1990 |
| Glutamate | 42163^a^  [38201, 46125] | 48961^b^  [46307, 51615] | 37800^c^  [34359, 41241] | 41134^a^  [39284, 42984] | C: 0.1149  AO: 0.9157 |
| **Remaining amino acids** | | | | | |
| Phenylalanine | 3038^a^  [2716, 3361] | 3835^b^  [3577, 4093] | 2969^a^  [2659, 3280] | 3678^b^  [3486, 3870] | C: 0.0750  **AO: 0.0263** |
| Tau-methylhistidine | 62.8  [56.1, 69.5] | 69.8  [63.9, 75.7] | 58.8  [51.9, 65.7] | 79.9  [75.0, 84.8] | C: 0.3525  AO: 0.2469 |
| Histidine | 3606^a^  [3265, 3948] | 4252^b^  [3999, 4505] | 3401^a^  [3089, 3712] | 3974^c^  [3791, 4157] | **C: 0.0157**  AO: 0.4039 |
| Hydroxyproline | 378.1^a^  [348.7, 407.5] | 477.1^b^  [450.7, 503.5] | 421.4^c^  [389.5, 453.2] | 578.6^d^  [555.2, 602] | **C: 0.0315**  AO: 0.4727 |
| Taurine | 1533^a^  [1431, 1634] | 1718^b^  [1645, 1790] | 2087^c^  [1955, 2218] | 2389^d^  [2311, 2467] | **C: <0.0001**  AO: 0.9585 |
| Tryptophan | 788^a^  [706, 870] | 1061^b^  [990, 1133] | 873^a^  [783, 964] | 1140^c^  [1082, 1197] | C: 0.3526  AO: 0.0740 |
| Data are presented as estimated mean [95% CI] in µmol/h. Statistics are by ANCOVA with multiple comparisons. ANCOVA was data as the dependent variable with confounders COPD, AO, COPD*AO interaction, gender, age, and BMI. ANCOVA p-values are effects of COPD, AO, or COPD*AO from the model. Interaction effects were tested for all variables with only significant effects being included; bold is p<0.05. Multiple comparisons are listed as superscript letters, same letters meaning no difference; q<0.05. Sum NEAA = Sum of the non-essential amino acids aspartate, glutamate, asparagine, glutamine, serine, glycine, arginine, alanine, proline and tyrosine. Sum EAA = Sum of the essential amino acids threonine, valine, methionine, isoleucine, leucine, tryptophan, phenylalanine, histidine and lysine. | | | | | |

| Additional file 1: Table S3. Whole body clearance estimates | | | | |
| --- | --- | --- | --- | --- |
|  | **COPD estimated difference vs. Control** | **p-values** | **AO estimated difference vs. non-AO** | **p-values** |
| **BCAA related amino acids** | | | | |
| Leucine | 142.3  [134.4, 150.1] | **0.0328** | -154.6  [-165.4, 143.7] | 0.4927 |
| Valine | 207.5  [-185.8, 229.1] | 0.2148 | 208.9  [-179.4, 238.4] | 0.4143 |
| Isoleucine | 54.7  [-44.1, 65.2] | 0.1025 | 62.5  [-47.8, 77.2] | 0.8981 |
| Glutamate | -1294.1  [-1655.9, 932.3] | 0.1216 | -1364.3  [-1858.1, 870.5] | 0.1573 |
| **Remaining amino acids** | | | | |
| Phenylalanine | -94.3  [-104.5, -84.1] | **0.0375** | 71.4  [-57.0, 85.8] | 0.0995 |
| Tau-methylhistidine | 0.2  [-3.2, 3.7] | 0.6719 | -3.3  [-8.0, 1.3] | 0.2396 |
| Histidine | -104.1  [-114.5, 93.8] | 0.0881 | 93.3  [-80.3, 106.3] | 0.7821 |
| Hydroxyproline | 10.7  [-6.4, 15.0] | 0.2239 | 11.3  [-5.6, 17.1] | 0.4891 |
| Taurine | 26.5  [15.0, 38.0] | **<0.0001** | -3.3  [-18.3, 11.8] | 0.6953 |
| Tryptophan | 10.4  [-1.9, 18.9] | 0.1279 | 11.8  [-0.4, 23.3] | 0.3735 |
| Data are presented as estimated differences [95% CI] from the COPD or AO effects in the ANCOVA model. ANCOVA was an amino acid WBP as the dependent variable with confounders gender, age, BMI, and amino acid concentration. ANCOVA p-values are effects of COPD or AO from the model; bold is p<0.05. Interaction effects were tested for all amino acids but none were significant. | | | | |

| Additional file 1: Table S4. Muscle and cognitive function | | | | | |
| --- | --- | --- | --- | --- | --- |
|  | **Non-AO Control  (n=52)** | **AO-Control**  **(n=116)** | **Non-AO COPD (n=65)** | **AO-COPD (n=134)** | **ANCOVA**  **p-values** |
| **Muscle function** | | | | | |
| Maximal leg extension torque (Nm) | 88.7^a^  [81.0, 96.4] | 92.4^a^  [86.9, 97.9] | 68.4^b^  [62.5, 74.2] | 75.0^c^  [71.6, 78.4] | **C: <0.0001**  AO: 0.2648 |
| Inspiratory muscle strength (cm H_2_O) | 85.9^a^  [82.1, 89.7] | 83.3^b^  [80.9, 85.8] | 48.6^c^  [46.3, 50.8] | 66.6^d^  [65.2, 68] | **C: <0.0001**  **AO: 0.0080**  **C*AO: 0.0040** |
| Expiratory muscle strength (cm H_2_O) | 102.2^a^  [97.5, 106.9] | 101.0^a^  [98.0, 104.0] | 63.9^b^  [60.8, 67] | 92.9^c^  [90.8, 95] | **C:<0.0001**  **AO: 0.0188**  **C*AO: 0.0047** |
| **Cognitive function** | | | | | |
| TMT - Pt A (Seconds) | 25.1^a^  [23.1, 27.2] | 26.6^a^  [25.8, 27.5] | 34.3^b^  [32.2, 36.4] | 36.2^c^  [35.1, 37.3] | **C: <0.0001**  AO: 0.2317 |
| TMT - Pt B (Seconds) | 54.8^a^  [49.7, 60] | 55.7^a^  [53.6, 57.9] | 78.7^b^  [72.2, 85.2] | 82.6^c^  [80.1, 85.2] | **C: <0.0001**  AO: 0.7867 |
| SCWT - Pt 1 (Seconds) | 46.4^a^  [45.7, 47] | 47.2^b^  [46.7, 47.7] | 53.9^c^  [53.1, 54.7] | 54.1^c^  [53.7, 54.5] | **C:<0.0001**  AO: 0.2086 |
| SCWT - Pt 2 (Seconds) | 54.3^a^  [54.0, 54.6] | 60.0^b^  [59.7, 60.4] | 63.6^c^  [63.2, 64.0] | 68.8^d^  [68.5, 69.2] | **C: <0.0001**  **AO: 0.0029** |
| SCWT - Pt 3 (Seconds) | 100.4^a^  [96.2, 104.6] | 105.8^b^  [103.4, 108.1] | 120.9^c^  [114.5, 127.2] | 125.3^d^  [122.6, 127.9] | **C: <0.0001**  AO: 0.2190 |
| SCWT INT (Seconds) | 48.7^a^  [44.6, 52.8] | 50.9^a^  [48.7, 53.1] | 60.8^b^  [53.8, 67.7] | 60.8^b^  [58.4, 63.2] | **C: 0.0038**  AO: 0.6658 |
| HADS: Depression | 2.0^a^  [2.0, 2.1] | 2.6^b^  [2.5, 2.6] | 4.0^c^  [3.8, 4.2] | 4.9^d^  [4.8, 5.0] | **C: <0.0001**  AO: 0.0835 |
| HADS: Anxiety | 3.0^a^  [2.8, 3.1] | 3.1^a^  [3.0, 3.2] | 5.1^b^  [4.9, 5.4] | 4.9^c^  [4.8, 5.0] | **C: <0.0001**  AO: 0.4325 |
| Data are presented as estimated mean [95% CI]. Statistics are by ANCOVA with multiple comparisons. ANCOVA was data as the dependent variable with confounders COPD, AO, COPD*AO interaction, gender, age, and BMI. ANCOVA p-values are effects of COPD, AO, or COPD*AO from the model. Interaction effects were tested for all variables with only significant effects being included; bold is p<0.05. Multiple comparisons are listed as superscript letters, same letters meaning no difference; q<0.05. TMT: Trail Making Test. SCWT: Stroop Color Word Test. SCWT INT: Stroop Color Word Test Interference. HADS: Hospital Anxiety and Depression Scale. | | | | | |
